# Impact of liver cirrhosis due to chronic hepatitis C viral infection on the outcome of ovarian cancer: a prospective study

**DOI:** 10.1186/s12885-015-1805-9

**Published:** 2015-10-21

**Authors:** Basel Refky, Sherif Kotb, Tamer Fady, Ahmad Marwan, Doaa Abd El-Khalek, Waleed Elnahas, Mohamed T. Hafez, Eduard Malik, Amr A. Soliman

**Affiliations:** Department of Surgical Oncology, Oncology Center Mansura University, University of Mansura, Mansura, Egypt; Transplant Center, Cleveland Clinic, Digestive Disease Institute, Cleveland, OH USA; Department of Public Health and Preventive Medicine, University of Mansura, Mansura, Egypt; Universitätsklinik für Gynäkologie und Geburtshilfe, Klinikum Oldenburg, Fakultät für Medizin und Gesundheitswissenschaften, Universität Oldenburg, Rahel-Straus-Straße 10, 26133 Oldenburg, Germany; Department of Obstetrics and Gynecology, University of Alexandria, Alexandria, Egypt

**Keywords:** Ovarian cancer, Liver cirrhosis, Surgery on hepatic patient

## Abstract

**Background:**

This study was designed to investigate the impact of liver cirrhosis due to chronic hepatitis C virus (HCV) infection on the disease-free and overall survival of ovarian cancer patients undergoing a standard primary operation followed by standard chemotherapy. Attainment of the operative goals, intra- and postoperative events, possible complications under chemotherapy necessitating the termination of treatment, and the impact of ovarian cancer treatment on liver function were assessed.

**Methods:**

This was a prospective observational study that included only patients with primary epithelial ovarian cancer. Only patients with Child-Turcotte-Pugh classification class A disease were recruited. Patients were divided into two groups according to whether they had liver cirrhosis. All the patients underwent primary debulking surgery followed by 6 cycles of chemotherapy, and were followed-up for 24 months after chemotherapy was completed.

**Results:**

We recruited 77 patients, 19 of whom had liver cirrhosis. There were no significant differences between patients with or without liver cirrhosis with respect to tumor stage, histopathological type, tumor grade, or optimal operative debulking. There was no registered liver dysfunction-related mortality in the follow-up period, and there were no statistically significant differences between the groups with respect to disease-free or overall survival (*p* = 0.719 and *p* = 0.524, respectively).

**Conclusion:**

From the results of this study, we conclude that compensated liver cirrhosis (Child-Turcotte-Pugh class A) due to chronic HCV infection affects neither the disease-free nor the overall survival of ovarian cancer patients, regardless of their stage. This study shows that it is possible to treat ovarian cancer patients with cirrhosis caused by HCV infection the same as any other patient; treatment does not have to be adjusted as long as the patients have Class A disease.

## Background

Ovarian cancer is the second most common genital cancer in women and has the highest mortality of all gynecological malignancies in developed countries [1]. In developing countries, ovarian cancer is the third most prevalent malignancy, and has the second highest mortality of all gynecological cancers [1]. Treatment of ovarian cancer consists of surgery aimed at complete tumor resection followed by 6 chemotherapy cycles of a combination of carboplatin and paclitaxel [2]. However, a number of other therapy regimens, including neoadjuvant chemotherapy followed by interval debulking for advanced primarily inoperable cases, have proven to be at least as effective [3].

Although the combined incidence rate of all cancers is nearly twice as high in more developed than in less developed countries in both men and women, the mortality rates of these cancers are only 8–15 % higher in more developed countries [1]. This discrepancy reflects the availability of advanced therapeutic technologies in more developed countries and the worse general condition of cancer patients in less developed countries.

The increasing life expectancy in many countries means that age, the presence of co-morbidities, and the patient’s general condition and performance status are increasingly important prognostic factors for survival [4–6], and health professionals have to care for many elderly ovarian cancer patients presenting with co-morbidities that may affect their treatment decisions [7].

Chronic hepatitis C virus (HCV) infection is a major burden on the health system in many developing countries including Egypt [8], where its prevalence in the 15–59 years old age group is as high as 14.7 % [9, 10], and thus considered to be the highest in the world [11, 12]. Approximately 50–85 % of patients with HCV infection develop chronic hepatitis, and of these, 20–30 % develop cirrhosis over a 20- to 30-year period [13]. HCV liver cirrhosis can be asymptomatic (compensated), presenting only as mild fatigue and subtle laboratory liver profile changes that are accidently discovered during a routine laboratory workup. However, it can also present in a decompensated, symptomatic form that reduces life expectancy. These symptoms can include ascites, variceal hemorrhage, encephalopathy, and hepato-renal syndrome, or it can develop over a number of years into hepatocellular carcinoma (HCC) [14]. Patients with liver cirrhosis currently undergo more surgical procedures compared to 20 or 30 years ago to because of improved post-operative survival secondary to improved methods of diagnosis and evolving therapies. Up to 10 % of cirrhotic patients might undergo surgery in the last 2 years of their lives [15]. Operating on a chronic liver patient poses a risk of liver injury due to perioperative drug administration along with alterations in hepatic blood flow caused by hemodynamic changes during anesthesia and surgery [16]. A widely acceptable tool to assess the perioperative risk in relation to liver function and the patient’s general condition is the Child-Turcotte-Pugh classification (Tables 1 and 2) [17]. Patients with good hepatic reserve, no ascites, and a good nutritional status (Child class A) can withstand surgery, whereas those with jaundice, low serum albumin, ascites, and muscle wasting have a higher operative mortality and postoperative morbidity [18].

**Table 1:** Child-Turcotte-Pugh Scores with respect to different clinical parameters [17]

	1 point	2 points	3 points
Total bilirubin, μmol/L (mg/dL)	<34 (<2)	34–50 (2–3)	>50 (>3)
Serum albumin, g/dL	>3.5	2.8–3.5	<2.8
INR	<1.7	1.71–2.30	>2.30
Ascites	None	Mild	Moderate to severe
Hepatic encephalopathy	None	Grade I-II (or suppressed with medication)	Grade III-IV (or refractory)

**Table 2:** Child-Turcotte-Pugh Scores with respect to different clinical parameters [17]

Points	Class	One-year survival	Two-year survival
5–6	A	100 %	85 %
7–9	B	85 %	57 %
10–15	C	41 %	37 %

This, however, was not validated in patients with epithelial ovarian cancer. The impaired liver function and consequently increased perioperative risk in these patients may be worsened by the primary operative treatment and the consequent adjuvant chemotherapy that are recommended as standard of care for epithelial ovarian cancer patients. As stated above, presence of comorbidities is an important prognostic factor when dealing with epithelial ovarian cancer patients. Liver function impairment, even if mild in Child class A patients, may have an impact on survival and prognosis of ovarian cancer patients. This was not previously investigated in published studies.

This study investigates the prognosis of epithelial ovarian cancer patients, in terms of overall and disease-free survival, who concomitantly suffer from liver cirrhosis due to chronic HCV infection and are class A according to Child-Turcotte-Pugh classification. The achievement of operative goals, intra- and postoperative adverse events, possible chemotherapy-related complications leading to the termination of treatment, and the impact of ovarian cancer treatment on liver function in these patients are also assessed.

## Methods

This is a prospective observational cohort study conducted in the Oncological Surgery Department, Oncology Center Mansura University, Mansura, Egypt. The study was approved by the institutional review board; the Ethics Committee, Faculty of Medicine, University of Mansura. (approval number 256/2010). The patient cohort included patients suffering from epithelial ovarian cancer, a subgroup of which suffers from liver cirrhosis due to chronic HCV infection and are exclusively classified as Child class A. All patients presenting with symptoms consistent with ovarian cancer, and who were being prepared for a primary explorative laparotomy between February 2011 and February 2013, gave signed, informed consent for participation. The final decision as to whether a patient should be included in the study was based on the final histopathology report. Only patients with epithelial primary ovarian cancer and a performance status of 0–2 according to the Eastern Cooperative Oncology Group classification were included [19]. Patients assigned to receive neoadjuvant treatment were excluded from this study, as were those younger than 18 years or older than 75 years. A routine preoperative abdominal and pelvic computed tomography scan was obtained. This was also used to identify patients with liver cirrhosis. CT criteria suggestive of liver cirrhosis include; heterogeneous liver parenchyma, surface nodularity in addition to the presence of signs of early portal hypertension, namely clinically silent small esophageal varices and caudate lobe hypertrophy [20, 21]. A laboratory liver profile was obtained, which included serum albumin, transaminases, and bilirubin levels, and the international normalization ratio (INR). Patients with liver cirrhosis from causes other than HCV infection were excluded from the study, as were liver cirrhosis patients with a Child-Turcotte-Pugh classification other than class A. After obtaining the final histopathology report, primary epithelial ovarian cancer patients were recruited for the study and divided into two groups: those with hepatic cirrhosis Child class A due to chronic HCV infection (group A) and those without cirrhosis or any detectable hepatic disorder (group B). All the patients received the institutional standard therapy for ovarian cancer, namely primary operative cytoreduction including explorative laparotomy, abdominal hysterectomy with bilateral salpingo-ovariectomy, omentectomy, peritoneal sampling, pelvic and para-aortic systematic lymphadenectomy, and eventually tumor debulking in patients with advanced stage disease through visceral organ resection and ultimately liver resection, as recommended by the German workgroup for gynecological oncology (AGO) for treating epithelial ovarian cancer [2]. All the patients received 6 cycles of chemotherapy postoperatively using combined paclitaxel (175 mg/m^2^ body surface area) and carboplatin (area under the curve [AUC], 5), both administered intravenously every 3 weeks as a standard adjuvant therapy, as recommended by the AGO [2]. Treatment was considered complete after the completion of chemotherapy. Operative data (operative time, amount of intra operative bleeding, visceral organ resection, liver resection, intraoperative urological and vascular complications and length of stay in the intensive care unit), the postoperative liver profile, and the development of postoperative complications were all recorded. Urological complications refer to bladder and/or ureteral injuries that are repaired intraoperatively. Vascular complications refer to venous or arterial injuries to the pelvic vessels and/or the vena cava or aorta that were immediately repaired intra-operatively with no further consequences. All the patients were then followed-up for 24 months after completing chemotherapy. Patients lost to follow-up were excluded from the statistical analysis. Follow-up data (late postoperative complications, chemotherapy complications, chemotherapy interruption, recurrence, liver function, and liver function-related complications) were also registered.

The sample size of the cohort (77) was calculated at a study power of 80 % and a level of significance at 5 %. The obtained data was tabulated using Microsoft Excel (Microsoft Corporation, Redmond, WA, USA) and analyzed using SPSS for Microsoft Windows, version 21.0 (SPSS, Chicago, IL, USA). The normality of data was first tested with the one-sample Kolmogorov-Smirnov test. Qualitative data are described using the number and percent. Continuous variables are presented as the mean ± standard deviation (SD) for parametric data, and the range and the median for non-parametric data. All the following tests are two tailed. Associations between categorical variables were tested using the Chi-square test, and groups were compared using the Student *t*-test (parametric data) or the Mann–Whitney *U* test (non-parametric data). The Kaplan-Meier test was used for survival analysis and the statistical significance of differences between curves was determined using the log-rank test. For all of the statistical tests, the threshold of significance was set at 5 % (p-value). Differences were considered significant if the probability of error was less than 5 % (p < 0.05). The confidence interval (CI) was set at 95 % for all tests of significance concerning disease-free as well as overall survival.

## Results

We recruited 77 patients who met the inclusion criteria between February 2011 and February 2013. Nineteen patients (24.7 %) had liver cirrhosis due to HCV infection (group A) while the other 58 patients (76.3 %) were free from any documented liver disease (group B). There was no significant difference between the groups with respect to age (*p* = 0.623); the median age was 58 years (range, 49–69 years) in group A and 57 years (range, 47–68 years) in group B. There were also no significant differences between the groups with respect to tumor stage, histopathological type, tumor grade, or optimal operative debulking (as defined by the AGO [2]) (Table 3). Likewise, both groups showed similar urological and vascular operative complications. None of the patients underwent liver resection. Liver capsule resection was not considered liver resection per se and was instead included with visceral organ resection. Visceral organ resection in turn refers to a range of procedures including intestinal resection with re-anastomosis or with colostomy/ileostomy, and partial bladder resection with or without ureteric re-implantation. The mean length of stay in the intensive care unit was not significantly different between the two groups (Table 4), and neither was the incidence of postoperative complications such as intestinal fistula or leakage, wound complications, lymphorrhea or lymphocysts, or thrombo-embolic events (Table 5). Preoperative serum albumin level was statistically significant lower in group A than in group B, [mean ± SD in mg/dL, group A = 3.59 ± 0.28, group B = 3.96 ± 0.39, (group A 3.2–3.76, group B 3.57–4.46, CI = 95 %), *p* ≤ 0.001]. Postoperative levels of serum albumin in group B were statistically significant lower than preoperative levels, [mean ± SD in mg/dL, preoperative = 3.96 ± 0.39, postoperative = 3.63 ± 0.46, (group A 3.56–4.39, group B 3.13–4.01, CI = 95 %), *p* ≤ 0.001]. Serum bilirubin level was statistically significant higher postoperatively in group A in comparison with group B [mean ± SD in mg/dL, group A = 1.11 ± 0.21, group B = 0.97 ± 0.16, (group A 0.99–1.29, group B 0.89–1.11, CI = 95 %) *p* = 0.004] and in comparison with preoperative levels in group A [mean ± SD in mg/dL, preoperative =0.963 ± 0.11, postoperative = 1.11 ± 0.21, (preoperative 0.871–0.999, postoperative 0.981–1.23, CI = 95 %) *p* = 0.008].

**Table 3:** Comparison between the groups with respect to FIGO stage, histopathological type, tumor grade, and operative cytoreduction

		Group A [*n* = 19 (24.7 %)]		Group B [*n* = 58 (75.3 %)]		
Tumor stage (FIGO)		I	9	I	19	*p* = 0.35
		II	5	II	10	
		III	5	III	27	
		IV	0	IV	2	
Histopathological type	Serous	17		48		*p* = 0.707
	Mucinous	1		6		
	Clear cell	1		4		
Tumor grade		G^1^	5	G^1^	18	*p* = 0.613
		G^2^	3	G^2^	11	
		G^3^	11	G^3^	29	
Operative cytoreduction		R0	14	R0	41	*p* = 0.802
		R1 and/or R2	5	R1 and/or R2	17

**Table 4:** Intra-operative procedures and complications, and the length of stay in the intensive care unit in each group

	Group A [*n* = 19 (24.7 %)]	Group B [*n* = 58 (75.3 %)]	
Operative time, minutes (mean ± SD)	202.42 ± 66.09	209.16 ± 60.03	*p* = 0.680
Intra-operative bleeding, ml (mean ± SD)	463 ± 76	485 ± 85	*p* = 0.49
Urological complications	1	5	*p* = 0.63
Vascular complications	2	4	*p* = 0.6
Visceral organs resection	4	20	*p* = 0.62
Length of stay in intensive care unit, days (median and range)	4	4	*p* = 0.229
	range 3–7	range 1–5

**Table 5:** Comparison of postoperative complications between the groups

	Group A [*n* = 19 (24.7 %)]	Group B [*n* = 58 (75.3 %)]	
GIT fistula or leakage	1	5	*p* = 0.63
Wound complications	1	7	*p* = 0.39
Lymphorrhea or lymphocyst	0	2	*p* = 0.41
Thrombo-embolic events	1	3	*p* = 1

AST, ALT and INR values did not statistically significant differ between both groups neither pre- nor post-operatively. These findings had no further significant impact on completion of treatment.

Only 1 patient had to discontinue chemotherapy in group A, and 2 in group B, due to intolerance to adverse reactions (neuropathy), although this difference was not statistically significant (*p* = 0.89).

**Fig. 1:**
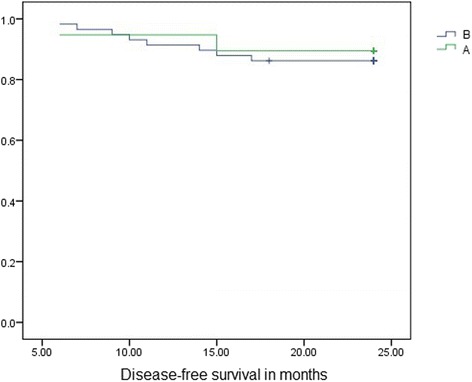
Kaplan-Meier curve showing disease-free survival in group A and B (*p* = 0.719, CI = 95 %). (**a** = group A, with liver cirrhosis. **b** = group B, with no liver cirrhosis)

**Fig. 2:**
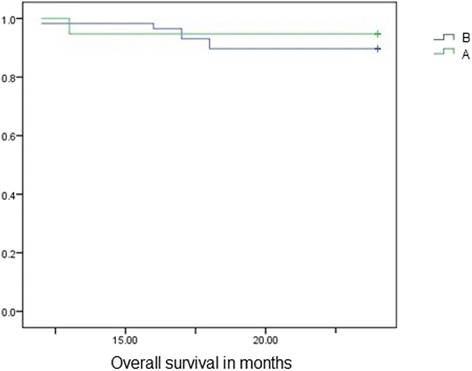
Kaplan-Meier curve showing overall survival in group A and B (*p* = 0.524, CI = 95 %). (**a** = group A, with liver cirrhosis. **b** = group B, with no liver cirrhosis)

During the follow-up period, 2 patients in group A and 8 in group B experienced recurrences, with 1 cancer-related death in group A and 7 in group B, although these were not related to liver dysfunction. Figures 1 and 2 show the disease-free and overall survival curves in both groups, respectively, using Kaplan-Meier estimators. There were no statistically significant differences between the groups with respect to disease-free or overall survival [disease-free survival was in group A 19.9–26.7 months (CI 95 %) and in group B 19.4–26.1 months (CI 95 %), while overall survival was in group A 21.6–25.7 months (CI 95 %) and in group B 21.1–25.1 months (CI 95 %), (*p* = 0.710 and *p* = 0.524, respectively)].

## Discussion

This is the first prospective study to investigate the relationship between liver cirrhosis caused by HCV infection and ovarian cancer. Our findings revealed that there was no significant impact of liver cirrhosis due to HCV infection on the disease-free or overall survival of ovarian cancer patients. Patients with liver cirrhosis showed some significant but minimal differences in their laboratory parameters that did not affect their liver condition or their capacity to complete adjuvant chemotherapy.

Our findings are in agreement with the published outcomes of liver cirrhosis patients with Child class A cirrhosis who underwent operative procedures. In those patients, the operative risk of elective surgery was moderate and surgical indications were not altered by the presence of cirrhosis [22, 23]. Our results confirm this outcome in ovarian cancer patients.

It seems that ovarian cancer patients with liver Child class A cirrhosis due to chronic HCV infection perform better than ovarian cancer patients with diabetes mellitus who generally have a worse prognosis compared to non-diabetic patients [6, 24, 25]. Ovarian cancer patients with liver cirrhosis have a better performance status than ovarian cancer patients with other co-morbidities that have a negative impact on survival [26, 27] and that affected the treatment decisions made by health care professionals, even leading to the discontinuation of treatment in some cases [28].

This study was limited by the small number of recruited patients and the extent to which laboratory liver parameters could be investigated. These limitations are attributed to the limited resources of the institution and the country in which the study took place.

## Conclusions

From the results of this study, we conclude that compensated liver cirrhosis (Child-Turcotte-Pugh class A) due to chronic HCV infection affects neither the disease-free nor the overall survival of ovarian cancer patients, regardless of their stage. This study shows that it is possible to treat ovarian cancer patients with cirrhosis caused by HCV infection the same as any other patient; treatment does not have to be adjusted as long as the patients have Class A disease.

## Abbreviations

HCV: Hepatitis C virus
HCC: Hepatocellular carcinoma
CT: Computer tomography
INR: International normalization ratio
AGO: German workgroup for gynaecological oncology
AUC: Area under the curve
SD: Standard deviation
CI: Confidence interval
AST: Aspartate transaminase
ALT: Alanine Transaminase
Mg: Milligram
dL: Deciliter
FIGO: International Federation of Obstetrics and Gynecology
